# Portal Vein Stenting Combined with Iodine-125 Seeds Endovascular Implantation Followed by Transcatheter Arterial Chemoembolization for Treatment of Hepatocellular Carcinoma Patients with Portal Vein Tumor Thrombus

**DOI:** 10.1155/2016/3048261

**Published:** 2016-11-24

**Authors:** Jun-Hui Sun, Tanyang Zhou, Tongyin Zhu, Yuelin Zhang, Chunhui Nie, Jing Ai, Guanhui Zhou, Aibin Zhang, Meng-Jie Dong, Wei-Lin Wang, Shu-Sen Zheng

**Affiliations:** ^1^Department of Hepatobiliary and Pancreatic Interventional Center, The First Affiliated Hospital, School of Medicine, Zhejiang University and Key Laboratory of Combined Multi-Organ Transplantation, Ministry of Public Health, Hangzhou, Zhejiang 310003, China; ^2^Department of Ophthalmology, The Second Affiliated Hospital, School of Medicine, Zhejiang University, Hangzhou, Zhejiang 310009, China; ^3^Department of Nuclear Medicine, The First Affiliated Hospital, School of Medicine, Zhejiang University, Hangzhou, Zhejiang 310003, China

## Abstract

Aim was to assess the therapeutic value of portal vein stenting (PVS) combined with iodine-125 seed (^125^I seed) strand endovascular implantation followed by transcatheter arterial chemoembolization (TACE) for treating patients with hepatocellular carcinoma (HCC) and portal vein tumor thrombus (PVTT). This was a retrospective study of 34 patients aged 29–81 years, diagnosed HCC with PVTT, and treated with PVS combined with ^125^I seed strand endovascular implantation followed by TACE between January 2012 and August 2014. Survival, stent patency, technical success rate, complications related to the procedure, and adverse events were recorded. The technical success rate was 100%. No serious procedure-related adverse event was recorded. The median survival was 147 days. The cumulative survival rates and stent patency rates at 90, 180, and 360 days were 94.1%, 61.8%, and 32.4% and 97.1% (33/34), 76.9% (24/34), and 29.4% (10/34), respectively. PVS combined with ^125^I seed strand endovascular implantation followed by TACE is feasible for patients with HCC and PVTT. It resulted in appropriate survival and stent patency, with no procedure-related adverse effects.

## 1. Introduction

Hepatocellular carcinoma (HCC) is one of the most common malignant tumors worldwide. It is particularly prevalent in China and has become the leading cause of cancer-related deaths among men in rural areas and second in urban areas [1]. HCC has a great propensity to invade the portal venous system and to induce portal vein tumor thrombus (PVTT). Indeed, PVTT is present in 44% of patients with HCC according to autopsy data and in 31–34% according to clinical data [2]. PVTT can cause partial or total portal vein occlusion and extensive intra- or extrahepatic metastases [3]. Moreover, PVTT in the main portal trunk puts pressure on the portal vein, which would further lead to gastrointestinal bleeding and ascites and induce multiple intrahepatic tumor dissemination and recurrence. If untreated, the prognosis of patients with HCC and PVTT becomes extremely poor [4]. The median survival of patients with HCC and PVTT is 2.7–4.0 months, whereas survival in patients with HCC but without PVTT is 24.4 months [5–7]. For these patients, there is no effective treatment and the optimal treatment remains controversial [3]. Most HCCs with PVTT are technically unresectable and they are not suitable for curative therapies. Palliative treatments include transcatheter arterial chemoembolization (TACE) and portal vein chemotherapy, percutaneous ethanol injection, ^125^I seed implantation, and laser ablation.

As a palliative treatment, TACE treatment for HCC with PVTT is safe and effective when there is sufficient collateral circulation [8, 9]. However, PVTT limits the effect of TACE on HCC and has a strong negative impact on the therapeutic effect [10]. Moreover, TACE will inevitably embolize some part of the blood supplies of normal liver tissues and increase the risk of liver failure. On the other hand, the portal vein pressure is rising with development of PVTT, which increases the risk of esophageal and gastric bleeding and indirectly leads to death [11]. Therefore, opening the occlusion of the portal vein caused by PVTT and reperfusing the portal vein could improve the success of TACE, and this could be achieved using portal vein stenting (PVS) [12–14]. PVS combined with TACE has been successfully used in the treatment of HCC with PVTT [6]. However, PVS effectively removes the portal vein obstruction but do not treat the thrombus per se, and stent restenosis may happen [6].

Radioactive seed implantation is used in a variety of solid tumors. ^125^I seed implantation was attempted to treat HCC with PVTT and achieved excellent therapeutic efficacy [15] with a good safety profile [16]. The combination of radiation therapy and TACE has been explored before [17, 18]. Zhang et al. [16] evaluated percutaneous puncture ^125^I seed implantation combined with TACE for HCC with PVTT under CT-guidance and showed a median survival of 18 months. Wu et al. [19] conducted PVS implantation and TACE combined with endovascular implantation of ^125^I seeds to treat HCC with PVTT and prolonged the survival of patients and the period of stent patency. However, only a few data are available about the combined therapeutic strategy of PVS and TACE combined with endovascular implantation of ^125^I seeds for treating patients with HCC and PVTT.

Therefore, this study was conducted to further explore the treatment efficacy of PVS combined with endovascular implantation of ^125^I seed strand followed by TACE for patients with HCC and PVTT. The results could provide new clues about the optimal therapeutic approaches for these patients.

## 2. Materials and Methods

### 2.1. Study Design

This retrospective study was conducted between January 2012 and August 2014 at the First Affiliated Hospital, School of Medicine, Zhejiang University, to evaluate the therapeutic value of PVS combined with ^125^I seed strand endovascular implantation followed by TACE for treating patients with HCC and PVTT. This study was approved by the Ethics Committee of the First Affiliated Hospital, School of Medicine, Zhejiang University. The need for individual consent was waived by the committee because of the retrospective nature of the study. The principles of the Declaration of Helsinki and Good Clinical Practice Guidelines were strictly followed.

### 2.2. Patients

34 eligible patients diagnosed with HCC and PVTT were included in this study. Inclusion criteria were as follows: (a) clinical diagnosis of HCC with PVTT (established by history, tumor markers, hepatitis series, imaging, and/or pathology); (b) tumor thrombi invading the portal vein, but at least one of the main branches of the portal vein was free; (c) Child-Pugh Class A or B; (d) absence of distant metastasis; (e) normal coagulation function (prothrombin time ≤17.0 s); (f) Karnofsky performance status (KPS) score ≥60; and (g) given informed clinical consent to the treatment. Exclusion criteria were as follows: (a) life expectancy <3 months; (b) coagulation disorders that could not be corrected; (c) widespread metastases; or (d) massive ascites.

The diagnosis of HCC was made according to the criteria of the Chinese Anti-Cancer Association [22]. PVTT was confirmed by ultrasound, CT, and/or MRI, and the classification was according to the Barcelona Clinic Liver Cancer (BCLC) staging [23]. The Liver Cancer Study Group of Japan suggested a macroscopic classification for PVTT, which categorized PVTT into five grades: (1) Vp0, no PVTT; (2) Vp1, presence of PVTT not in, but distal to, the secondary order branches of the portal vein; (3) Vp2, presence of PVTT in the 2nd-order branches of the portal vein; (4) Vp3, presence of PVTT in the 1st-order branches of the portal vein; and (5) Vp4, presence of PVTT in the main trunk of the portal vein or a portal vein branch contralateral to the mainly involved lobe (or both).

### 2.3. Procedures

Endovascular implantation of ^125^I seed strands was performed as previously described [15]. Conventional chemoembolization was performed using doxorubicin (Pfizer, New York, NY, USA) mixed with 5–20 mL of iodized oil (Lipiodol Ultra-Fluide, Laboratoire Guerbet, Villepinte, France). The dose of doxorubicin was 50–75 mg/m^2^, adjusted according to the patient's liver function and body surface area. Super selection of the tumor feeding artery was performed using a microcatheter. The mixture was infused at a rate of 0.5–1 mL/min until blood flow stasis in tumor vascularity was achieved under a fluoroscope. Gelatin sponge (Jingling, Nanjing, China) was used to embolize the feeding artery of the tumor. Artery-portal vein shunt, if present, was embolized by polyvinyl alcohol or Embosphere according to the angiography prior emulsion infusion.

### 2.4. Stenting and ^125^I Seed Implantation

Nitinol self-expandable stents (Luminexx III, Bard Peripheral Vascular, Inc., Tempe, AZ, USA, or Smart Control, Cordis Medical, Fremont, CA, USA), with a diameter of 12–14 mm and length of 60–100 mm, were routinely used. Model 6711 ^125^I seeds (Seeds Biological Pharmacy Ltd., Tianjin, China) were enveloped in a 3 Fr sterile plastic tube (Create Medic, Tokyo, JAP). The diameter and length of the titanium capsule are 0.8 mm and 4.5 ± 0.5 mm. The radioactivity of each ^125^I seed is 25.9 MBq with a half-life of 59.4 days. The main photon emissions are 27.4 and 31.4 keV X-rays and 35.5 keV *γ*-rays. The half-value thickness of tissue for ^125^I seeds is 17 mm, and the initial dose rate is 7 cGy/h. The effective irradiation range is 20 mm.

After local anesthesia, the patent 2nd-order branch of the intrahepatic portal vein was punctured transhepatically with an 18-gauge Chiba needle (Cook Medical, Bloomington, IN, USA) under ultrasound guidance. When access to the portal vein was confirmed, a 0.035-inch wire (Cook Medical, Bloomington, IN, USA) was manipulated into the superior mesenteric vein. A 6-F NEFF set (Cook Medical, Bloomington, IN, USA) was introduced into the portal vein over the 0.018-inch wire. After wire exchange, the NEFF set was replaced by a 5-F sheath (Cordis Medical, Fremont, CA, USA). Portography was performed by a 5-F calibrated pigtail catheter (Cook Medical, Bloomington, IN, USA) placed in the superior mesenteric vein. The diameter and length of the obstructed MPV were measured. The number of ^125^I seeds planned to be implanted was calculated by the following formula: *N* = length of obstructed MPV (mm)/4.5 + 2. These seeds were arranged linearly and sealed into a 3-F sterile plastic tube continuously to construct a ^125^I seed strand. Two 0.035-inch diameter and 260 cm long stiff wires (Terumo, Tokyo, Japan) were inserted into the superior mesenteric vein through the 5-F sheath. After the sheath was removed, the outer cannula of the 6-F NEFF set and the stent with appropriate size were introduced in the MPV over one of the stiff wires. The stent was deployed from distal MPV into proximal patent intrahepatic portal vein. The ^125^I seed strand was inserted into the target position through the outer cannula of the NEFF set. When the NEFF set was withdrawn, the radioactive seed strand was released and fixed steadily between the stent and MPV. Portography was performed again. Finally, the transhepatic puncture track was occluded by coils with diameters of 3–5 mm.

**Figure 1:**
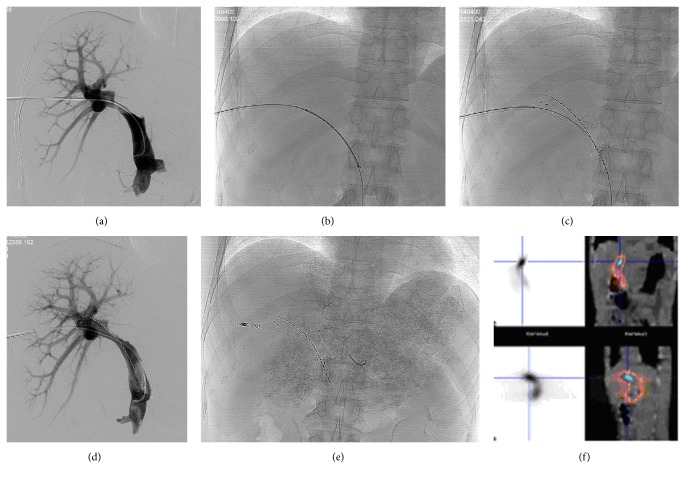
Images of ^125^I seed strand implantation in a 53-year-old man. (a) After the patent 2nd-order branch of the left portal vein was catheterized, a 5-F Cobra catheter was placed in the main portal vein (MPV). Tumor thrombus in the proximal MPV and sagittal segment of the left portal vein was clearly shown in the right anterior oblique portography projection. (b) Transcatheter implantation of ^125^I seed strand. (c) Under guidance of the second guide wire, the portal vein stent was implanted. (d) Angiography after vein stent and ^125^I seed strand implantation showing the portal vein blood flow and that the seeds position is good. (e) A 14 × 120-mm self-expandable stent and ^125^I seed strand with 20 seeds were placed precisely in the obstructed MPV. The ^125^I seed strand was fixed steadily between the stent and MPV. Good flow through the patent stent from distal MPV to left portal vein is shown on the portography. (f) Images from single-photon emission computed tomography (SPECT)/computed tomography (CT) 1 day after procedure. The stent and ^125^I seed strand were placed correctly, without displacement. Radiation emitted by the ^125^I seed strand was distributed homogeneously. It presented as a cylindrical shape with a diameter of 20 mm covering the target lesion completely.

Among the 34 patients, 34 stents and 36 ^125^I seed strands (750 ^125^I seeds) were endovascularly implanted; two patients with a large PVTT were implanted with two ^125^I seed strands (of 30 and 40 seeds, resp.) (Figure 1).

### 2.5. TACE

To identify all feeding arteries of the tumor, angiography of the celiac, hepatic, superior mesenteric, left gastric, and bilateral inferior phrenic arteries was performed using a 5-F RH catheter (Cook Medical, Bloomington, IN, USA). The target artery was catheterized with a 2.7-F microcatheter (Terumo, Tokyo, Japan). The drugs were oxaliplatin 150–200 mg, HCPT 10–20 mg, pirarubicin 10–20 mg, and lipiodol (Laboratoire Guerbet, Villepinte, France). If necessary, embolization particles such as PVA particles (Alicon, Inc., Hangzhou, China), one kind of microspheres called Embosphere (Biosphere Medical Inc., USA), or small particles of gelatin sponge were used to strengthen embolization. The embolization extent was determined according to the tumor size and patients' liver function.

### 2.6. Postoperative Management and Follow-Up

After the procedure, all patients received supportive liver protection therapy for at least 3 days and anticoagulation treatment with heparin and biaspirin for 1–3 months [24]. Analgesics were prescribed if necessary. Antiviral drugs were prescribed for patients with hepatitis B. All patients underwent abdominal CT the day after the procedure. Routine laboratory test data (liver function, renal function, and blood cell count and coagulating parameter) from the first and third days after the procedure were collected.

Repeat TACE with the same protocol was performed if residual tumor was visible with enhancement on arterial phase or occurrence of a new lesion. Indirect portography was routinely performed to evaluate the patency of the stent. If decompensation or decline of liver function in clinical status occurred, repeated chemoembolization was not performed. All patients were followed up until death or November 2015. Stent patency was defined as the ratio of in-stent restenosis <50% [25]. If the ratio of in-stent restenosis was ≥50%, follow-up was terminated and the number of days with stent patency was recorded.

During follow-up, the 34 patients received 78 TACE treatments including one patient of five treatments, four patients with four treatments, eight patients with three treatments, 12 patients with two treatments, and nine patients with a single treatment.

### 2.7. Statistical Analysis

Continuous data are presented as mean ± SD. Categorical data are presented as frequencies. Survival and stent patency analysis was performed with the Kaplan-Meier method. All statistical analyses were performed using SPSS 17.0 (IBM, Armonk, NY, USA).

## 3. Results

### 3.1. Characteristics of the Patients

Table 1 presents the characteristics of the 34 patients included. Age range was 29–81 years (median 54 years). The Child-Pugh classification was A in 23 patients and B in 11. Twenty-four patients had multiple scattered lesions and 10 had a single lesion. Twelve patients were with right portal vein tumor thrombus, five with left, 13 with main and right, and four with main and left.

Among these patients, 31 patients achieved TACE treatment within 1 week, and the other three patients experienced transient hepatic dysfunction after PVS combined with endovascular implantation of the ^125^I seed strand and underwent TACE 10 days later.

### 3.2. Adverse Effects

After symptomatic treatments, all patients achieved technical success, for a technical success rate of 100%. Technique-related complications such as puncture bleeding, bile leak, liver abscess, abdominal hemorrhage, tumor bleeding, gastrointestinal bleeding, or other serious complications did not occur during follow-up.

### 3.3. Survival and Stent Patency

**Figure 2:**
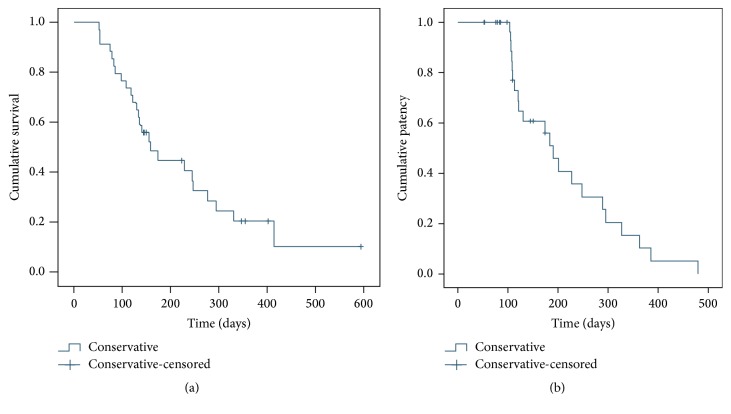
(a) Survival curve of patients with HCC and PVTT after stent and ^125^I seed strand implantation and TACE. (b) Stent patency over time in patients with HCC and PVTT after stent and ^125^I seed strand implantation and TACE.

Follow-up ended in November 2015. The median survival time was 147 days. The 90-, 180-, and 360-day cumulative survival rates were 94.1%, 61.8%, and 32.4% (Figure 2(a)). At the end of follow-up, 27 patients had died. The causes of death were lung metastases (*n* = 3), gastrointestinal bleeding (*n* = 8), liver failure (*n* = 13), brain metastases (*n* = 1), vertebral metastases (*n* = 1), and heart failure (*n* = 1).

The 90-, 180-, and 360-day stent patency rates were 97.1% (33/34), 76.9% (24/34), and 29.4 (10/34) (Figure 2(b)).

## 4. Discussion

Only a few data were available about the combined therapeutic strategy of PVS and TACE combined with endovascular implantation of ^125^I seeds for treating patients with HCC and PVTT. Therefore, this study was conducted to further explore the treatment efficacy of PVS combined with endovascular implantation of ^125^I seed strand followed by TACE for patients with HCC and PVTT. Results showed that the technical success rate of the treatment was 100%. No serious procedure-related adverse events were recorded. The cumulative survival and stent patency rates at 90, 180, and 360 days were 94.1%, 61.8%, and 32.4% and 97.1% (33/34), 76.9% (24/34), and 29.4 (10/34), respectively.

Radioactive seed implantation for the treatment of solid tumors is well established, especially in prostate tumors [20]. Long half-life (*t*_1/2_ 59.43 days) and short distance from the tumor tissues make ^125^I seed and the *γ*-rays emitted by the seeds inhibit vascular endothelial hyperplasia, prolonging the time of stent patency [13].

The first reported treatment modality that was directly targeting the PVTT using an interventional radiology technique was arterial chemoembolization combined with transportal ethanol injection in 1999 [21]. Thereafter, Zhang et al. [16] showed that ^125^I seed implantation combined with TACE for HCC with PVTT under CT-guidance greatly improved the rates of biliary and vascular injury. In the present study, patients were treated with a ^125^I seed strand prepared using the appropriate number of ^125^I seeds within a 3-F sheath tube according to the length of the PVTT based on portal vein angiography. This approach avoided biliary and vascular injury caused by repeated puncture. Moreover, with the expansion of the stent, the ^125^I seeds strand is fixed in the site of tumor thrombus, which prevents iodine-125 seeds loss or displacement. In the present study, all patients were successfully treated.

Zhang et al. [14] analyzed the therapeutic results of percutaneous transhepatic PVS combined with TACE in 58 patients with HCC and PVTT; they observed that the 180- and 360-day cumulative survival was 27.1% and 17.2%, respectively. In the present study, the 180- and 360-day cumulative survival was 61.8% (21/34) and 32.4% (11/34), higher than that of the treatment of PVS combined with TACE [6, 14]. However, the 180- and 360-day stent patency rates were 76.9% (24/34) and 29.4% (10/34) in the present study, which were slightly lower than in previous studies (71.0% and 52.6%) [14]. Differences in population and in the criteria for restenosis might explain a part of the discrepancy. Meanwhile the severity of PVTT has been reported to be important prognostic factor. And, to some extent, patients with PVTT classified into Vp4 in our study occupied large proportion, which might be another reason that leads to the discrepancy.

In the present study, the stent patency in six patients with Child-Pugh B liver function was lower than that in patients with Child-Pugh A function. It might suggest that the liver function may affect the prognosis of patients with HCC at the same stage. Of course, no conclusion could be reached on this observation, but it could provide clues for future studies.

This study had some limitations. The sample size was small and from a single hospital, preventing subgroup and detailed analyses of the factors influencing treatment success. In addition, the retrospective nature of the study prevented assessing factors that were not routinely collected. The follow-up time was short but because of the poor prognosis of these patients, it was sufficient to observe mortality events. Finally, there was no control group. Additional studies are necessary to assess adequately the use of this treatment approach.

In conclusion, PVS combined with ^125^I seed strand endovascular implantation followed by TACE is feasible for patients with HCC and PVTT. It resulted in appropriate survival and stent patency, with no procedure-related adverse effects.

## Figures and Tables

**Table 1:** Characteristics of patients.

Characteristics	
Age, years, median (range)	54 (29–81)
Sex (male/female)	32/2
Number of lesions	
Single	10
Multiple	24
Location of tumor thrombosis	
LPVB	5
RPVB	12
MPV + LPVB	4
MPV + RPVB	13
Child-Pugh class	
A	23
B	11
Degree of PVTT, *n* (%)	
Vp2	6 (18)
Vp3	11 (32)
Vp4	17 (50)

LPVB: left portal vein branch; RPVB: right portal vein branch; MPV: main portal vein; PVTT: portal vein tumor thrombus.

The Liver Cancer Study Group of Japan suggested a macroscopic classification for PVTT, which categorized PVTT into five grades: (1) Vp0, no PVTT; (2) Vp1, presence of PVTT not in, but distal to, the 2nd-order branches of the portal vein; (3) Vp2, presence of PVTT in the 2nd-order branches of the portal vein; (4) Vp3, presence of PVTT in the 1st-order branches of the portal vein; and (5) Vp4, presence of PVTT in the main trunk of the portal vein or a portal vein branch contralateral to the mainly involved lobe (or both).

## References

[1] Shi J., Lai E. C. H., Li N. (2011). A new classification for hepatocellular carcinoma with portal vein tumor thrombus. *Journal of Hepato-Biliary-Pancreatic Sciences*.

[2] Kim J.-Y., Chung S.-M., Choi B.-O., Kay C.-S. (2011). Hepatocellular carcinoma with portal vein tumor thrombosis: improved treatment outcomes with external beam radiation therapy. *Hepatology Research*.

[3] Poddar N., Avezbakiyev B., He Z., Jiang M., Gohari A., Wang J. C. (2012). Hepatocellular carcinoma presenting as an incidental isolated malignant Portal vein thrombosis. *Journal of Gastrointestinal Cancer*.

[4] Zhou L., Rui J.-A., Wang S.-B., Chen S.-G., Qu Q. (2013). Risk factors of poor prognosis and portal vein tumor thrombosis after curative resection of solitary hepatocellular carcinoma. *Hepatobiliary & Pancreatic Diseases International*.

[5] Lin D.-X., Zhang Q.-Y., Li X., Ye Q.-W., Lin F., Li L.-L. (2011). An aggressive approach leads to improved survival in hepatocellular carcinoma patients with portal vein tumor thrombus. *Journal of Cancer Research and Clinical Oncology*.

[6] Luo J., Guo R.-P., Lai E. C. H. (2011). Transarterial chemoembolization for unresectable hepatocellular carcinoma with portal vein tumor thrombosis: A Prospective Comparative Study. *Annals of Surgical Oncology*.

[7] Takayasu K., Arii S., Ikai I. (2006). Prospective cohort study of transarterial chemoembolization for unresectable hepatocellular carcinoma in 8510 patients. *Gastroenterology*.

[8] Kim K. M., Kim J. H., Park I. S. (2009). Reappraisal of repeated transarterial chemoembolization in the treatment of hepatocellular carcinoma with portal vein invasion. *Journal of Gastroenterology and Hepatology*.

[9] Shi M., Chen J.-A., Lin X.-J. (2010). Transarterial chemoembolization as initial treatment for unresectable hepatocellular carcinoma in southern China. *World Journal of Gastroenterology*.

[10] Liu L., Zhang C., Zhao Y. (2014). Transarterial chemoembolization for the treatment of advanced hepatocellular carcinoma with portal vein tumor thrombosis: prognostic factors in a single-center study of 188 patients. *BioMed Research International*.

[11] Jiang Z.-B., Shan H., Shen X.-Y. (2004). Transjugular intrahepatic portosystemic shunt for palliative treatment of portal hypertension secondary to portal vein tumor thrombosis. *World Journal of Gastroenterology*.

[12] Luo J.-J., Zhang Z.-H., Liu Q.-X., Zhang W., Wang J.-H., Yan Z.-P. (2016). Endovascular brachytherapy combined with stent placement and TACE for treatment of HCC with main portal vein tumor thrombus. *Hepatology International*.

[13] Gong G., Wang X., Wang J. (2007). The portal venous pressure change duo to metallic stents implanted into portal veins in HCC patients. *Journal of Interventional Radiology*.

[14] Zhang X.-B., Wang J.-H., Yan Z.-P., Qian S., Liu R. (2009). Hepatocellular carcinoma invading the main portal vein: treatment with transcatheter arterial chemoembolization and portal vein stenting. *CardioVascular and Interventional Radiology*.

[15] Yang M., Fang Z., Yan Z. (2014). Transarterial chemoembolisation (TACE) combined with endovascular implantation of an iodine-125 seed strand for the treatment of hepatocellular carcinoma with portal vein tumour thrombosis versus TACE alone: a two-arm, randomised clinical trial. *Journal of Cancer Research and Clinical Oncology*.

[16] Zhang F.-J., Li C.-X., Jiao D.-C. (2008). CT guided 125iodine seed implantation for portal vein tumor thrombus in primary hepatocellular carcinoma. *Chinese Medical Journal*.

[17] Dae Y. K., Park W., Do H. L. (2005). Three-dimensional conformal radiotherapy for portal vein thrombosis of hepatocellular carcinoma. *Cancer*.

[18] Salem R., Lewandowski R. J. (2013). Chemoembolization and radioembolization for hepatocellular carcinoma. *Clinical Gastroenterology and Hepatology*.

[19] Wu L. L., Luo J. J., Yan Z. P. (2012). Comparative study of portal vein stent and TACE combined therapy with or without endovascular implantation of iodine-125 seeds strand for treating patients with hepatocellular carcinoma and main portal vein tumor thrombus. *Zhonghua gan Zang Bing za Zhi*.

[20] Rossi P. J. (2015). Role of brachytherapy for advanced prostate cancer. *Current Problems in Cancer*.

[21] Yamakado K., Nakatsuka A., Tanaka N., Matsumura K., Takase K., Takeda K. (1999). Long-term follow-up arterial chemoembolization combined with transportal ethanol injection used to treat hepatocellular carcinoma. *Journal of Vascular and Interventional Radiology*.

[22] (2000). The diagnostic criterias of primary liver cancer from Chinese Society of Liver Cancer. *Journal of Hepatology*.

[23] Pons F., Varela M., Llovet J. M. (2005). Staging systems in hepatocellular carcinoma. *HPB*.

[24] Nio Y., Iguchi C., Itakura M. (2009). Placement of an expandable metallic stent improves the efficacy of chemoradiotherapy for pancreatic cancer with malignant portal vein stenosis or obstruction. *Anticancer Research*.

[25] Yang P., Chen Z., Jiang J., Zhang Y.-Y., Zhou H., Wu Q.-H. (2010). The analysis to risk factors of affecting the long-term patency ratio of stent planted in arteriosclerosis subclavian artery. *Zhonghua Yi Xue Za Zhi*.

